# An advanced hybrid deep learning framework for high-precision brain tumor detection and classification in MRI scans

**DOI:** 10.1038/s41598-026-50194-x

**Published:** 2026-04-29

**Authors:** Basu Dev Shivahare, Shamala K. Subramaniam, Dafik, Sunder R., Sangeetha S. K. B., Siva Shankar S.

**Affiliations:** 1 School of Computing Science and Engineering, Galgotias University, Greater Noida, Uttar Pradesh 203201 India; 2 Department of Communication Technology and Network, Faculty of Computer Science and Information Technology, University Putra Malaysia (UPM), Serdang, Selangor 43400 Malaysia; 3 Department of Mathematics, Faculty of Mathematics and Natural Sciences, University of Jember, Jember, Indonesia; 4 Manipal Institute of Technology Bengaluru, Manipal Academy of Higher Education, Manipal, India; 5 Department of Computer Science and Engineering, KG Reddy College of Engineering and Technology, Hyderabad, Telangana 501504 India

**Keywords:** Brain tumor detection, Deep learning framework, Multi-scale CNN, Semi-supervised learning, Self-adjusting attention mechanism, MRI classification, Cancer, Computational biology and bioinformatics, Engineering, Health care, Mathematics and computing, Medical research, Oncology

## Abstract

Early and accurate identification of brain tumors from magnetic resonance imaging (MRI) is essential for timely clinical intervention; however, manual interpretation remains time-consuming and dependent on expert analysis. The study proposes MultiAttenNet, a hybrid deep learning framework that integrates multi-scale convolutional neural networks (CNNs) for hierarchical feature extraction and Transformer-based attention mechanisms for global contextual learning, within a semi-supervised learning paradigm. The multi-scale feature extraction improves robustness in detecting tumors of varying sizes and irregular structures, while the adaptive attention module dynamically emphasizes diagnostically relevant regions to enhance localization and reduce false positives. A consistency-based semi-supervised learning scheme enables effective training using limited labeled data alongside unlabeled samples, improving generalization across diverse clinical scenarios. The framework is evaluated using the BraTS 2023 for glioma segmentation and publicly available datasets such as the Figshare Brain Tumor Dataset for multi-class tumor classification (glioma, meningioma, and pituitary tumors), MultiAttenNet achieves accuracy, sensitivity, specificity, and false-positive rates of 98.4, 96.8, 99.2 and 1.3%, respectively, outperforming existing state-of-the-art approaches. The proposed framework provides a scalable and efficient solution for real-time clinical brain tumor diagnosis and supports reliable automated decision-making in neuro-oncology.

## Introduction

The clinical diagnosis, therapy planning, and patient survival are dependent on the early and correct detection of brain tumors by means of Magnetic Resonance Imaging (MRI) techniques. The correct identification of tumors by MRI modalities is difficult, even with the availability of high-resolution imaging techniques, such as the size, shape, position, and intensity of tumors differ from patient to patient. The correct computer-aided solution is needed, as the manual diagnosis of tumors by radiologists is tedious, subjective, and sensitive to inter-observer differences.

With recent advancements in deep learning techniques, particularly through Convolutional Neural Networks (CNNs), brain tumor analysis has improved through end-to-end feature learning directly from images. The ability of CNNs to effectively conduct brain tumor segmentation and classification has demonstrated through various architectures, such as U-Net, ResNet, and DenseNet. Various problems with brain tumor analysis using existing techniques remain. First, existing CNN-based brain tumor analysis techniques perform poorly when dealing with small or irregularly shaped tumors because they cannot simultaneously obtain local tumor features and global context information. The reliance on large datasets due to the costly labeling of medical pictures is another problem with brain tumor analysis using CNNs. The problem of false positives and unstable localization is caused by the fixed attention mechanism’s incapability to adapt to changing tumor features.

Even though recent hybrid and transformer-based approaches try to address the issue of contextual learning, the applicability of these approaches in real-time clinical environments is limited due to their high computational complexity and requirement of a large amount of labeled data. Generalization under different MRI acquisition scenarios remains quite challenging. This highlights the need for a scalable system to learn from sparse labeled data, extract strong features, and deal with flexible regions.

The study proposes MultiAttenNet, a hybrid deep learning framework for brain tumor identification and classification, as a solution to these problems. The proposed framework includes a self-adjusting attention mechanism, semi-supervised learning, and multi-scale convolutional feature learning. The proposed methodology aims to improve the accuracy of brain tumor localization and reduce false detections through the simultaneous learning of multi-scale features and attention. The proposed approach also incorporates consistency-based semi-supervised learning, further enhancing generalization performance using labeled and unlabeled images. The proposed approach aims to provide an accurate, efficient, and viable solution for neuro-oncology diagnostics.

The main contributions are.

1. To develop MultiAttenNet, a hybrid architecture that integrates multi-scale convolutional neural networks to capture both global contextual information and fine-grained tumor structural details from MRI scans.
2. To introduce a Transformer-based dynamic attention module that adaptively focuses on diagnostically relevant tumor regions, enhancing feature representation and reducing false-positive predictions.
3. To incorporate a consistency-regularized semi-supervised learning framework that effectively utilizes both labeled and unlabeled MRI data, improving generalization and reducing dependency on manual annotations.

## Related study

With the help of advancements in deep learning, hybrid models, and explainable artificial intelligence (XAI), the problem of automated identification of brain tumors using Magnetic Resonance Imaging (MRI) has gained much attention. Convolutional neural networks, hybrid learning methods, optimization techniques, and data fusion techniques are the major techniques used to improve the accuracy of the classification process. The following methodological issues are unclear.

CNN-based architectures for tumor classification were presented. To enhance fine-grained representation learning, the hybrid CNN framework(h-CNN)^1^, used multi-resolution feature extraction. Although the accuracy of this model was enhanced, its capability to learn long-range contextual relationships within MRI volumes was limited by its reliance on locality, which is a characteristic of CNN. DenseNet-based models^2^ and deep learning-based tumor classification techniques^3,4^ demonstrated feature learning capabilities; but interpretability was not incorporated.

Explainable AI-based models were proposed as a solution to such transparency-related issues. CNN models and interpretability modules were combined in a hybrid explainable model in^5^, which generated explanations for predictions in a visual form. Techniques in attention-based learning were further employed to highlight key locations of tumors in a localization technique in^6^. These models often increased computational complexity without optimizing classification and efficiency, even when they improved interpretability.

Strategies for hybrid and ensemble learning have been extensively explored. The improvement of robustness through variety in models has been demonstrated in various experiments that combine CNN models and SVM classifiers^7^, decision tree classifiers^8^, and even ensemble deep networks such as the Xception and CNN model combination^9^. When applied in real-world scenarios, such models have been observed to have issues of scalability and require complex training procedures.

The efficiency of convergence was enhanced using particle swarm optimization techniques, quantum-inspired optimization, and Bayesian optimization^10,11^. Instead of overcoming the issues of representation, which are commonly found in CNN structures, these optimization techniques have focused on the maximization of parameters. To reduce the scarcity of labeled medical data, transfer learning is used extensively^12–14^. Domain shift between natural and medical images results in decreased relevance of features, which highlights the importance of domain adaptive architectures despite using transfer learning for better generalization.

In the pursuit of effective global contextual linkages, the hybrid models of Transformer and CNN have been emphasized in the recent studies^15,16^. Though the models based on the Transformer topology can improve the modeling of long-range dependencies, the applicability of the models to the clinical environment with limited resources is restricted due to the considerable computing complexity. To overcome the problem, the CNN models^17^ with limited complexity often sacrifice the richness of the representation. For enhancing robustness and protecting privacy, techniques based on multi-modal and federated learning have been introduced. In the context of protecting the privacy of patients, there is an improvement in the cross-institutional generalization with the help of multi-modal fusion techniques^18^, ensemble learning frameworks^19,20^, and federated learning frameworks with weighted aggregation^21,22^. Instead of integrating privacy, interpretability, and efficiency into a single framework, most of the existing works treat all these aspects as separate.

By utilizing residual connections and attention mechanisms to improve the localization of the boundary and feature representation, a modified model of the recurrent residual attention U-Net architecture was proposed to achieve the precise segmentation of tumors; however, it is associated with increased complexity during training^23^. A computer-aided diagnostic system utilizing CNN-based feature extraction and transfer learning was proposed to achieve multi-class classification of brain tumors ^24^. The model exhibited improved performance even when trained on small datasets; however, it was dependent on pre-trained feature representations^25^. Has proposed a framework that utilizes deep feature-based models to integrate segmentation and classification techniques to achieve improved diagnostic performance.

Furthermore, recent advancements in the study of explainability methods utilize diffusion-based augmentation methods^26^, fair learning approaches^27^, Vision Mamba topologies for efficient long-range modeling^28,29^, and SHAP-based methods^30^. Although these methods enhance various aspects, such as efficiency or fairness, there is still much to be explored in a system that incorporates interpretability, semi-supervised learning, efficient optimization, and multi-resolution learning. Table 1 shows the comparison of existing brain tumor detection methods.

**Table 1:** Comparison of existing brain tumor detection methods.

Ref	Method	Dataset Type	Task	Strength	Limitation
^1^	h-CNN	MRI	Classification	Multi-resolution features	Limited global context
^5^	XAI-CNN	MRI	Multi-class classification	Interpretability	High computational cost
^7^	MobileNetV2 + SVM	MRI	Classification	Hybrid prediction	Pipeline complexity
^9^	Xception Ensemble	MRI	Classification	Improved robustness	High training cost
^18^	Multimodal Fusion DL	Multi-modal MRI	Classification	Data fusion	Requires multiple modalities
^6^	Attention DL	MRI	Classification	Better localization	Limited generalization
^13^	Transfer Learning CNN	MRI	Classification	Works with small data	Domain mismatch
^15^	CNN–Transformer	MRI + Genomic	Detection	Global context modeling	Computationally expensive
^21,22^	Federated Learning	Distributed MRI	Training	Privacy preservation	Communication overhead
^30^	SHAP-based XAI	MRI	Explainability	Transparency	No accuracy improvement
^28,29^	Vision Mamba	MRI	Classification	Efficient long-range learning	Early-stage research

## Research gap

From the above analysis, the following limitations are identified: CNN-based models lack global contextual understanding, Transformer models improve context learning but increase computational complexity, Explainable models often compromise efficiency, Hybrid systems rarely integrate semi-supervised learning for limited medical datasets, Privacy-preserving and interpretable learning are seldom unified within a single architecture.

### Motivation of proposed work

To address these challenges, the proposed framework integrates Multi-scale CNN feature extraction, Transformer-based attention for global dependency modeling, Semi-supervised learning for label efficiency, Optimization-driven training strategies, Interpretable attention mechanisms for clinical transparency. This unified design aims to achieve improved classification accuracy, scalability, and explainability suitable for real-world clinical decision support systems.

## System methodology

The Brain Tumor Segmentation (BraTS) 2023 dataset is used in this study for glioma-focused analysis. The BraTS dataset is a widely adopted benchmark in medical image analysis and consists exclusively of glioma cases, including high-grade glioma (HGG) and low-grade glioma (LGG). It provides multi-parametric MRI (mpMRI) scans, comprising four modalities: T1-weighted (T1), T1-weighted post-contrast (T1c), T2-weighted (T2), and Fluid Attenuated Inversion Recovery (FLAIR). These modalities offer complementary information for accurate tumor characterization and segmentation.

Each MRI scan in the BraTS dataset is accompanied by expert-annotated segmentation masks, which delineate tumor subregions, including:

- Enhancing tumor (ET)
- Tumor core (TC)
- Whole tumor (WT), including edema

The dataset is primarily designed for 3D volumetric segmentation tasks, where each subject consists of co-registered MRI volumes with a standard resolution of 240 × 240 × 155 voxels. The annotations enable precise localization and volumetric analysis of glioma regions (Table 2).

**Table 2:** Summary of datasets used in this study.

Dataset	Tumor Type	No. of Subjects	Modalities	Task
BraTS 2023	Glioma (HGG, LGG)	~ 1,250 patients	T1, T1c, T2, FLAIR	Segmentation
Figshare Brain Tumor Dataset	Glioma, Meningioma, Pituitary	3,064 images	MRI	Classification

It is important to note that the BraTS dataset does not include meningioma or pituitary tumor classes. Therefore, for multi-class tumor classification involving glioma, meningioma, and pituitary tumors, additional publicly available datasets (e.g., Figshare and Kaggle brain tumor datasets) are utilized. These datasets provide labeled MRI images corresponding to distinct tumor categories and are commonly used for classification tasks.

In this study:

- The BraTS 2023 dataset is used for glioma segmentation and evaluation of tumor region detection.
- The Figshare/Kaggle brain tumor datasets are used for multi-class classification experiments involving glioma, meningioma, and pituitary tumors.

The dataset is divided into training, validation, and testing subsets following standard protocols. Data augmentation techniques such as rotation, flipping, and scaling are applied to improve model generalization and robustness. All methods and procedures in this study were conducted in accordance with relevant ethical standards. The datasets used are publicly available, anonymized, and de-identified, and therefore do not involve direct human subject participation. The study complies with ethical guidelines for medical imaging research.

### Preprocessing

The preprocessing stage is designed to handle multi-modal MRI data (T1, T2, and FLAIR), where each modality provides complementary diagnostic information.

Initially, all MRI images are resized to a uniform resolution (256 × 256) to ensure computational consistency. Since different MRI modalities exhibit varying intensity distributions, modality-specific normalization is performed:

Normalization:

1 $${\mathrm{I}}_{{{\mathrm{normalized}}}}^{{\mathrm{m}}} \left( {{\mathrm{x}},{\mathrm{y}}} \right) = {\mathrm{I}}^{{\mathrm{m}}} \left( {{\mathrm{x}},{\mathrm{y}}} \right)/{\mathrm{max}}\left( {{\mathrm{I}}^{{\mathrm{m}}} } \right)$$

where *m* $\in$ *{T1, T2, FLAIR}* denotes the modality.

To further standardize the data, z-score normalization is applied independently for each modality:

2 $${\mathrm{I}}_{{{\mathrm{standardized}}}}^{{\mathrm{m}}} \left( {{\mathrm{x}},{\mathrm{y}}} \right) = \left( {{\mathrm{I}}^{{\mathrm{m}}} \left( {{\mathrm{x}},{\mathrm{y}}} \right) - \mu^{{\mathrm{m}}} } \right)/\sigma^{{\mathrm{m}}}$$

where μ^m^ and σ^m^ represent the mean and standard deviation of the respective modality.

To reduce noise while preserving structural details, Gaussian filtering is applied:

3 $${\mathrm{I}}_{{{\mathrm{filtered}}}} {\mathrm{m}}\left( {{\mathrm{x}},{\mathrm{y}}} \right) = \sum {\sum {{\mathrm{I}}^{{\mathrm{m}}} \left( {{\mathrm{x}} + {\mathrm{i}},{\text{ y}} + {\mathrm{j}}} \right)} } \cdot {\mathrm{G}}\left( {{\mathrm{i}},{\mathrm{j}}} \right)$$

Following preprocessing, the modalities are aligned and combined into a multi-channel input representation:

4 $${\mathrm{I}}_{{{\mathrm{multi}}}} = \left[ {{\mathrm{I}}_{{{\mathrm{T1}}}} ,{\mathrm{I}}_{{{\mathrm{T2}}}} ,{\mathrm{I}}_{{{\mathrm{FLAIR}}}} } \right]$$

This multi-channel representation enables the model to learn complementary features across modalities. To improve generalization, data augmentation techniques such as rotation, flipping, and scaling are applied consistently across all modalities to maintain spatial alignment. The dataset is split into training, validation, and test sets (70:15:15). To address class imbalance, class-weighted loss functions are employed, assigning higher importance to underrepresented classes. These preprocessing steps ensure that the multi-modal MRI data is normalized, aligned, and enriched, enabling effective learning for tumor detection and classification.

### Proposed framework

**Fig. 1:**
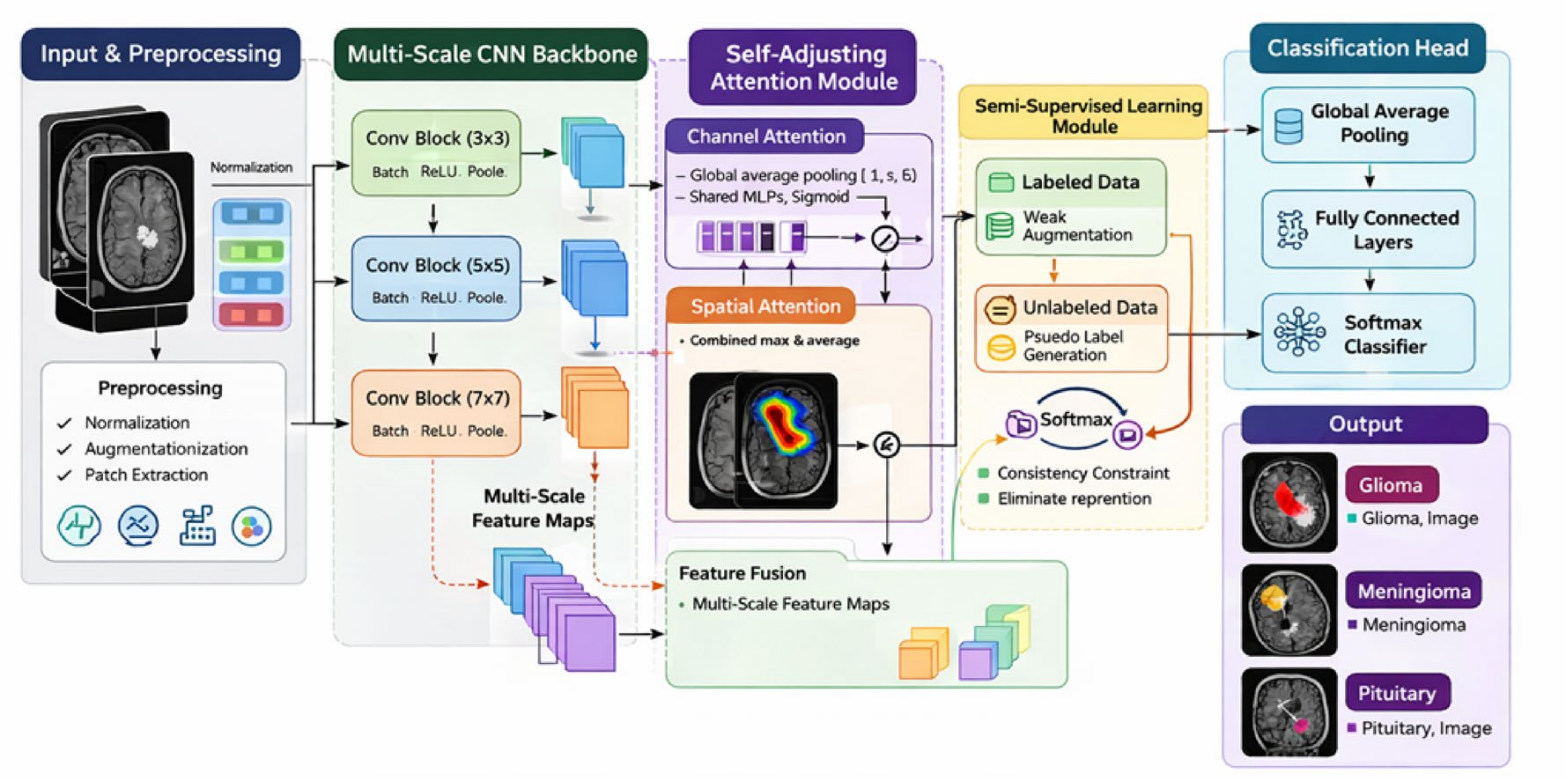
Proposed MultiAttenNet Framework integrating multi-scale CNN feature extraction, Transformer-based attention mechanism, and semi-supervised learning module.

MultiAttenNet is a unified hybrid architecture consisting of three key components: (i) a multi-scale CNN module for extracting spatial features at different receptive fields, (ii) a Transformer-based attention module for modeling long-range dependencies and refining feature representations, and (iii) a semi-supervised learning module for leveraging both labeled and unlabeled MRI data. These components are integrated into a single end-to-end trainable framework. The method focuses on multi-class brain tumor classification using MRI slices. While segmentation-inspired mechanisms are incorporated to improve spatial feature representation, the final output of the model is a class label rather than a segmentation mask. With enhanced real-time processing and diminished dependence on large manually annotated datasets, this method aims at improving robustness, accuracy, and generalizability of brain tumor detection. Figure 1 shows the proposed framework.

The model performs multi-class tumor classification using 2D MRI slices as input, yielding one of three output labels: pituitary, meningioma, or glioma. The architecture uses a hybrid design in which hierarchical and geographically rich features are first extracted by multi-scale CNN layers. These features are then fused and serially sent to a Transformer-based attention module. To efficiently capture long-range contextual dependencies throughout the image, the fused feature maps are specifically molded into token sequences and processed utilizing multi-head self-attention. U-Net is modified for classification by adding a global pooling layer and a fully connected classification head to guarantee a fair comparison with baseline models. This change makes it possible to use classification metrics for consistent evaluation.

During attention processing, the structural integrity of medical images is maintained via positional encoding obtained from convolutional feature maps, which preserves spatial information. The overall methodology uses global context modeling from Transformers and local feature extraction from CNNs to create a hybrid architecture that improves discriminative capabilities without requiring explicit segmentation output. The suggested Transformer-based attention captures long-range dependencies, which are essential for precisely detecting complex tumor structures, in contrast to conventional attention mechanisms like channel or spatial attention (e.g., SE and CBAM).

#### Multi-scale convolutional neural networks (CNNs) for feature extraction (MultiAttenNet)

The MultiAttenNet, utilizes a multi-branch convolutional structure, which enables the efficient extraction of fine-grained, mid-level, and global context data. The proposed model utilizes the concept of simultaneous multi-scale feature extraction, which enables the efficient representation of tumors of various sizes, shapes, and textures, as opposed to the sequential processing of data as done in conventional CNN models. The input MRI image is first processed through the multi-scale CNN branches, followed by feature fusion and refinement using Transformer-based attention, and finally optimized using a semi-supervised learning strategy for classification and localization.

The proposed model utilizes three convolutional branches, each of which processes the input MRI slice after it has been scaled up to the size of 256 × 256 × 1:

- Fine-scale branch: Utilizes 3 × 3 kernels with 32 filters to capture high-frequency details such as tumor edges, boundaries, and micro-textures. This branch focuses on precise localization of small and irregular tumor regions.
- Medium-scale branch: Employs 5 × 5 kernels with 64 filters to extract intermediate structural patterns, including tumor shape and surrounding tissue variations.
- Coarse-scale branch: Uses 7 × 7 kernels with 128 filters to capture global contextual information, such as tumor position relative to overall brain anatomy.

Each branch consists of two convolutional layers, where each convolution is followed by Batch Normalization and ReLU activation to improve training stability and non-linearity. A max-pooling layer (2 × 2) is applied at the end of each branch to reduce spatial dimensionality while preserving salient features.

##### Feature fusion strategy

A channel-wise concatenation technique is used to merge the feature maps from the three branches, creating a combined feature tensor that preserves multi-scale data. After concatenation, a 1 × 1 convolution layer is applied, and then ReLU activation is used to improve feature interaction and decrease dimensional redundancy. The model can learn the best weighting across many scales using this process.

Formally, let ${F}_{f}$, ${F}_{m}$, and ${F}_{c}$ denote feature maps from fine, medium, and coarse branches, respectively. The fused representation is obtained as:

5 $$F_{fusion} = \sigma \left( {W_{1 \times 1} *\left[ {F_{f} \left\| {F_{m} } \right\|F_{c} } \right]} \right)$$

where $\mid \mid$ denotes concatenation, ${W}_{1\times 1}$ represents the learnable weights of the 1 × 1 convolution, and $\sigma$ is the ReLU activation function.

##### Hierarchical feature learning

For hierarchical abstraction, deeper convolutional layers get the fused feature representation. Higher-level semantic properties, such as tumor form, geographic distribution, and contextual relationships with neighboring tissues, are gradually learned by these layers. After feature fusion, a dropout layer (rate = 0.3) is added to enhance generalization and avoid overfitting. The Transformer-based attention module receives the final feature representation and refines it further.

#### Transformer-based attention mechanism for enhanced tumor localization

The model can avoid unnecessary background information and focus on the most relevant tumor sites by having Transformer-based attention mechanisms included. To ensure the model focuses on the relevant tumor sites, the self-attention mechanism is trained to dynamically assign weights to different MRI scan segments. When applied to complex images with noise or ambiguous regions, this approach addresses the issue of false positives, which is common in deep learning tumor diagnosis. By enabling the medical professionals to visualize what regions of the scan the model paid attention to, the attention mechanism enhances interpretability and increases confidence in the predictions. Deep learning models with Transformer-based attention mechanisms have revolutionized several fields, particularly those that deal with tasks of long-range interdependence and contextual understanding. The attention mechanism enhances the ability of the model to focus on relevant tumor regions and ignore irrelevant regions in the context of brain tumor detection from MRI scans. This enhances localization accuracy and addresses the issue of false positives.

##### Attention mechanism

Enabling the model to dynamically put different weights on different parts of the input image depending on their pertinence to the task at hand is the very essence of the attention mechanism. The model needs to downplay noise or distracting background areas in the MRI scan and keep its focus on the tumor location in order to identify brain tumors. The self-attention mechanism of the Transformer model handles this dynamic weighing process. The attention mechanism operates on the input feature map $F\in {R}^{H\times W\times C}$, where $H$, $W$, and $C$ are the height, width, width, and number of channels of the feature map, respectively. In the Transformer-based model, the feature map is transformed into query (Q), key (K), and value (V) matrices, which are used to compute the attention scores. The attention module uses H attention heads with embedding dimension D, where feature maps are flattened into T tokens before attention computation.

##### Self-attention mechanism

In a self-attention method, the network learns a corresponding query, key, and value for every pixel or region in the image. The main objective is to calculate attention scores, which establish the appropriate level of concentration for each component of the image when processing other components.

Given the input feature map $F$, we first linearly transform it into the query $Q$, key $K$, and value $V$ matrices.

6 $$Q = F \cdot W_{q} , K = F \cdot W_{k} , V = F \cdot W_{v}$$

where ${W}_{q},{W}_{k},{W}_{v}\in {R}^{C\times D}$ are learned weight matrices for the query, key, and value, respectively, $D$ is the dimension of the query, key, and value vectors.

The attention scores, which establish the relative importance of each spatial place in the image, are calculated using the query Q and key K. After scaling, these scores are calculated as the dot product of the query and key matrices.

7 $$Attention\,Scores = \frac{{Q \cdot K^{T} }}{\sqrt D }$$

where $\cdot$ denotes the matrix multiplication,${K}^{T}$ is the transpose of the key matrix, $\sqrt{D}$ is the scaling factor to prevent large values of the dot product.

The attention scores are then passed through a softmax function to obtain the normalized attention weights.

8 $$\alpha = Softmax\left( {\frac{{Q \cdot K^{T} }}{\sqrt D }} \right)$$

To guarantee that the attention weights are positive and add up to one for every row in the attention score matrix, the softmax function is applied to each row. The weighted sum of the value matrix V, which is the attention mechanism’s ultimate output, is calculated using these attention weights.

9 $$Output_{att} = \alpha \cdot V$$

The output ${Output}_{att}$ is a weighted sum of the values based on the computed attention weights, allowing the model to focus on the most relevant regions of the input image while ignoring non-relevant ones.

The term ‘self-adjusting attention’ refers to the adaptive reweighting of spatial features based on query-key similarity scores, enabling the model to dynamically emphasize diagnostically relevant regions while suppressing irrelevant background information.

##### Multi-head attention

Multi-head attention is a crucial component of Transformer models since it enables the model to recognize various kinds of linkages and contextual dependencies in various areas of the image. The model calculates several sets (or “heads”) of attention scores in parallel, each with a distinct learnt weight matrix, as opposed to calculating a single set. The final attention output is then created by concatenating and linearly transforming the outputs of these heads.

For multi-head attention, we define $H$ as the number of attention heads, and the attention operation is repeated $H$ times with different learned weight matrices ${W}_{q}^{h},{W}_{k}^{h},{W}_{v}^{h}$ for each head.

10 $$Q_{h} = F \cdot W_{q}^{h} , K_{h} = F \cdot W_{k}^{h} , V_{h} = F \cdot W_{v}^{h}$$

The attention scores for each head are computed as

11 $$\alpha_{h} = Softmax\left( {\frac{{Q_{h} \cdot K_{h}^{T} }}{\sqrt D }} \right)$$

The output for each head is then.

12 $$Output_{att}^{h} = \alpha_{h} \cdot V_{h}$$

Finally, the output of all heads are concatenated and linearly transformed to obtain the final multi-head attention output

13 $$Output_{multihead} = Concat\left( {Output_{att}^{1} ,Output_{att}^{2} , \ldots ,Output_{att}^{H} } \right) \cdot W_{o}$$

where ${W}_{o}\in {R}^{H\times D}$ is a learned output weight matrix that projects the concatenated attention outputs into the desired output dimension.

##### Enhancing tumor localization with attention

By assigning the tumor areas of the MRI scan large attention weights and minimizing the irrelevant background areas, the attention mechanism ensures the model focuses on the most relevant sections of the scan. By allowing the model to prioritize strongly discriminative tumor regions, this dynamic feature reweighting helps to improve localization precision. Depending on the content of the scan and its comparison to other regions, the self-attention mechanism assists the model dynamically focusing on different regions across the entire MRI scan. Particularly in complicated MRI scans with background noise or confusing regions where tumor-like characteristics could appear, this attention mechanism assists in reducing false positives.

To guarantee the boundaries and edges of the tumor are well localized, the attention mechanism assigns higher attention weights to regions which are spatially proximally and contextually relevant to the tumor when it identifies a tumor in a particular region of the image. The self-attention mechanism increases the accuracy of the tumor localization process by assisting the network in concentrating on areas with a high tumor likelihood and ignoring unimportant areas, including healthy brain tissue. The increased interpretability provided by Transformer-based attention mechanisms is an important advantage. The attention weights the model used can be viewed by medical staff, displaying where on the MRI scan the model found to be most relevant for tumor detection and classification. Frequently referred to as an attention map, this image supports physicians in understanding how the model reached its predictions.

##### Model interpretability and trust

The attention map can be visualized as a heatmap, with lower values indicating regions of the image with less attention and higher values indicating regions with greater attention. This enables medical practitioners to verify that the model is paying attention to the correct regions of the scan and have faith in its decision-making process. This enables healthcare providers to trust the model’s decision-making process and verify that it focuses on the correct regions of the scan

14 $$Attention\,Map = \alpha$$

This transparency is crucial in clinical settings, as it fosters confidence in AI-based predictions and facilitates collaboration between AI systems and medical professionals. Adding a self-attention mechanism based on a Transformer to the deep learning setup for brain tumor detection enhances the model’s ability to eliminate unwanted background information and focus on the most relevant tumor sites. To make sure that the model focuses on the tumor-related features and reduces the effect of background noise, the self-attention mechanism adjusts attention dynamically along the MRI scan. This approach addresses false positives and enhances localization accuracy and is an effective tool for enhancing brain tumor detection systems’ performance. Decision-making is explainable because attention mechanisms are interpretable, and therefore, physicians can verify and trust the model’s predictions.

#### Semi-supervised learning with consistency regularization for data efficiency

We propose to blend consistency regularization with a semi-supervised learning approach to bypass this issue. The model can learn from both labeled and unlabeled samples due to this method. The model can enhance its generalization ability without requiring intensive annotations using a large volume of unlabeled MRI data. Further enhancing resilience, consistency regularization ensures that the model is going to keep producing consistent predictions even when slight input perturbations are introduced. This approach allows the model to learn data from a greater range while requiring very little in the way of human labeling, which is quite valuable when dealing with imbalanced datasets like rare types of tumors.

Step 1: Initialize the Model and Dataset.

- Labeled Dataset: A small set of labeled MRI scans $L=\{({x}_{i},{y}_{i}){\}}_{i=1}^{{n}_{l}}$, where ${x}_{i}$ are the input images (MRI scans) and ${y}_{i}$ are their corresponding labels (tumor types, segmentation masks).
- Unlabeled Dataset: A large set of unlabeled MRI scans $U=\{{x}_{i}{\}}_{i=1}^{{n}_{u}}$, where the labels are unknown.

The model is initialized with random parameters $\theta$ (weights and biases of the neural network).

Step 2: The loss function for the labeled data in a supervised learning environment may be either a dice loss (for segmentation tasks) or a cross-entropy loss (for classification tasks). For a labeled dataset, the classification loss is

15 $$L_{supervised} = - \sum\limits_{i = 1}^{{n_{l} }} {y_{i} log(p(y_{i} |x_{i} ,\theta ))}$$

where $p({y}_{i}|{x}_{i},\theta )$ is the predicted probability distribution for the label ${y}_{i}$ given the input ${x}_{i}$ and model parameters $\theta$.

For segmentation tasks, we use the Dice loss

16 $$L_{dice} = 1 - \frac{{2\left| {\hat{y} \cap y} \right|}}{{\left| {\hat{y}} \right| + \left| y \right|}}$$

where $\hat{y}$ is the predicted segmentation mask, $y$ is the ground truth segmentation mask.

Step 3: The model generates reliable predictions even when the input data is somewhat perturbed using consistency regularization. We apply perturbations to the input images x, like random cropping, Gaussian noise, or random augmentations (like flipping or rotation). Both labeled and unlabeled data are subjected to these disturbances.

Let $\tilde{x}$ denote the perturbed version of $x$, which is generated by applying random augmentations to $x$

17 $$\tilde{x} = Augment\left( x \right)$$

The consistency regularization is to enforce that the model’s predictions for $x$ and its perturbed version $\tilde{x}$ are consistent. We define the consistency loss as the Mean Squared Error (MSE) between the model’s predictions for $x$ and $\tilde{x}$

18 $$L_{consistency} = \frac{1}{{n_{u} }}\sum\limits_{i = 1}^{{n_{u} }} {\left\| {f\left( {x_{i} ,\theta } \right) - f\left( {\tilde{x}_{i} ,\theta } \right)} \right\|}_{2}^{2}$$

where $f(x,\theta )$ is the model’s prediction for the input $x$, ${\| \cdot \| }_{2}^{2}$ denotes the squared ${L}_{2}$-norm (MSE loss), $\tilde{x}_{i}$ is the perturbed version of ${x}_{i}$.

Step 4: The consistency regularization term and the supervised loss are combined to form the total loss function. Both the consistency loss on unlabeled data and the supervised loss on labeled data are minimized during model training.

19 $$L_{total} = L_{supervised} + \lambda L_{consistency}$$

where $\lambda$ is a hyperparameter that controls the weight of the consistency regularization term in the total loss function.

For classification tasks, the total loss becomes:

20 $$L_{total} = - \sum\limits_{i = 1}^{{n_{l} }} {y_{i} log(p(y_{i} |x_{i} ,\theta ))} + \lambda \frac{1}{{n_{u} }}\sum\limits_{i = 1}^{{n_{u} }} {\left\| {f\left( {x_{i} ,\theta } \right) - f\left( {\tilde{x}_{i} ,\theta } \right)} \right\|}_{2}^{2}$$

For segmentation tasks, it becomes

21 $$L_{total} = L_{dice} + \lambda \frac{1}{{n_{u} }}\sum\limits_{i = 1}^{{n_{u} }} {\left\| {f\left( {x_{i} ,\theta } \right) - f\left( {\tilde{x}_{i} ,\theta } \right)} \right\|}_{2}^{2}$$

Step 5: Using the total loss ${L}_{total}$, the model’s parameters $\theta$ are updated via backpropagation and an optimization algorithm (such as Adam or SGD). The gradients of the total loss with respect to the model parameters are computed

22 $$\nabla_{\theta } L_{total} = \nabla_{\theta } L_{supervised} + \lambda \nabla_{\theta } L_{consistency}$$

The model parameters $\theta$ are updated as follows

23 $$\theta_{t + 1} = \theta_{t} - \eta \nabla_{\theta } L_{total}$$

where $\eta$ is the learning rate, ${\theta}_{t}$ is the parameter vector at iteration $t$.

Step 6: The model is trained iteratively over multiple epochs. In each epoch, the following steps occur.

- Select a batch from the labeled dataset $L$ and a batch from the unlabeled dataset $U$,
- Apply perturbations to the unlabeled data to generate $\tilde{x}_{i}$,
- Compute both the supervised loss for labeled data and the consistency loss for unlabeled data,
- Update the model parameters $\theta$ using the combined loss function.

This process continues until convergence, which is typically determined by monitoring the validation accuracy or loss.

24 $${\mathrm{F1}} = {\mathrm{Conv3}} \times {3}\left( {\mathrm{X}} \right)$$

25 $${\mathrm{F2}} = {\mathrm{Conv5}} \times {5}\left( {\mathrm{X}} \right)$$

26 $${\mathrm{F3}} = {\mathrm{Conv7}} \times {7}\left( {\mathrm{X}} \right)$$

27 $${\mathrm{F}}_{{{\mathrm{multi}}}} = {\mathrm{Concatenate}}\left( {{\mathrm{F1}},\,{\mathrm{F2}},\,{\mathrm{F3}}} \right)$$

28 $${\mathrm{F}}_{{{\mathrm{att}}}} = \sigma \left( {{\mathrm{Wc}}*{\mathrm{F}}_{{{\mathrm{multi}}}} } \right) \otimes {\mathrm{F}}_{{{\mathrm{multi}}}}$$

where σ = sigmoid, ⊗  = element-wise multiplication.

Attention weights enhance important regions and suppress irrelevant features.

29 $${\mathrm{L}}_{{{\mathrm{total}}}} = {\mathrm{L}}_{{{\mathrm{sup}}}} + \lambda \,{\mathrm{L}}_{{{\mathrm{unsup}}}}$$

where Supervised loss L_sup_ = CrossEntropy(y, y_pred_), Unsupervised loss (consistency), L_unsup_ =|| f(x)—f(x′) ||^2^.

Model learns from labeled data and enforces consistency on unlabeled data. For unlabeled samples, pseudo-labels are generated based on high-confidence predictions. These pseudo-labels are used as ground truth to further train the model, improving generalization.

To improve performance, generalization, and deployment of deep learning models for brain tumor detection from MRI scans, the proposed framework employs a variety of state-of-art methodologies. Some of the techniques used are: multi-class and multi-task learning, real-time inference optimization, data augmentation, and synthetic data generation. All these factors work together to form an efficient, interpretable, and effective model suitable for deployment in clinical environments.

#### Data augmentation and synthetic data generation for generalization improvement

By introducing simulated variation in tumor orientations, shapes, and image quality, these manipulations assist the model in learning to recognize tumors in any given situation. For the model to generalize better and to be more robust and operate with other MRI machines and patient populations, this data augmentation is applied. Further, the technique produces synthetic MRI images that capture uncommon or underrepresented tumor shapes through Generative Adversarial Networks (GANs). Through dataset balancing, the imitative images guarantee that the model is not biased against the more frequent tumor shapes. The addition of GAN-generated images augments diversity in the training set and enhances the model’s generalization across tumor types and MRI machines, which further boosts the performance of the model in real-world clinical setups.

#### Real-time inference with model optimization

The system is optimized by applying methods like model pruning, quantization, and the usage of light-weight network designs to achieve real-time processing. Model pruning decreases the model size without any degradation in accuracy by eliminating redundant or unnecessary parameters. By reducing the precision of the activations and weights, quantization further compresses the model and improves computation without degrading the model performance. Smaller models with less computational requirements and faster processing times are created with light architecture such as MobileNet. These advancements allow the model to apply to real-time tumor localization and classification, ensuring its adoption in high-speed clinical environments where immediate decision-making relies on fast processing of MRI data.

#### Multi-class and multi-task learning for comprehensive tumor analysis

The model uses a multi-task learning approach to address various domains of tumor analysis apart from generic tumor classification. The model can perform tasks like tumor segmentation and tumor classification into glioma, meningioma, pituitary tumor, etc., simultaneously because of multi-task learning. The ability of the model to learn multiple tasks simultaneously improves tumor classification and segmentation performance by being able to recognize more intricate relationships among the features of a tumor, like its type, position, and morphology. Tumor segmentation, for example, can be employed to find precise borders of a tumor, which is essential in coming up with treatment plans. Apart from this, the model is able to detect brain anatomy and tumor margins at the same time because of multi-task learning, which improves the accuracy of tumor detection and clinical decision-making. Through the combination of different aspects of tumor properties, the combined methodology makes the model able to provide a better diagnosis.

The envisioned framework builds a robust, effective, and trustworthy brain tumor diagnosis system from MRI scans using enhanced data augmentation, synthetic data generation, real-time optimization, and multi-task learning. With the help of these methods, the system can deal with unbalanced and heterogeneous datasets, provide real-time outputs, and give an exhaustive report of tumors, all while being transparent and assured due to explainable AI. Its practical application is supplemented by its smooth integration into clinical routines, which will pave the way for better decision-making and quicker, more accurate diagnosis in actual healthcare settings. The model is able to generalize across several MRI scans and uncommon tumor types because of the data augmentation in conjunction with the synthetic data produced by GAN, and real-time inference optimization enables it to be usable in clinical environments.

The complexity analysis of the proposed model reveals its computational demands, especially due to the integration of multi-scale CNN, Transformer-based attention, and semi-supervised learning. The time complexity of the multi-scale CNN is driven by factors such as the number of layers $L$, input size $N$, filter size $K$, and stride $S$. The time complexity for a single convolutional layer is $O({N}^{2}\cdot {K}^{2}\cdot {C}_{in}\cdot {C}_{out}/{S}^{2})$, where ${C}_{in}$ and ${C}_{out}$ are the input and output channels. For the entire CNN, considering $L$ layers and $R$ resolutions, the total time complexity becomes $O(L\cdot R\cdot {N}^{2}\cdot {K}^{2}\cdot {C}_{in}\cdot {C}_{out}/{S}^{2})$, and the space complexity for storing the outputs of the convolutional layers is $O(R\cdot {N}^{2}\cdot {C}_{out})$.

The Transformer-based attention mechanism has a time complexity proportional to the square of the sequence length $T$, which depends on the number of patches in the image. The time complexity for self-attention is $O({T}^{2}\cdot H)$, where $H$ is the hidden size. For the entire Transformer model, the total time complexity is $O(L\cdot A\cdot {T}^{2}\cdot H)$, where $A$ is the number of attention heads, and $L$ is the number of layers. The space complexity is $O(L\cdot T\cdot H)$. In semi-supervised learning, the time complexity for generating pseudo-labels for unlabeled data is $O({N}_{unlabeled}\cdot {T}_{model})$, where ${N}_{unlabeled}$ is the number of unlabeled samples and ${T}_{model}$ is the time complexity for a single forward pass through the model. Iterating this process for $I$ iterations lead to a total time complexity of $O(I\cdot {N}_{unlabeled}\cdot {T}_{model})$. The space complexity for storing the pseudo-labeled data is $O({N}_{unlabeled}\cdot {T}_{model})$.

The total time complexity of the model is the sum of the complexities of the CNN, attention mechanism, and semi-supervised learning, leading to

30 $$T_{total} = O\left( {L \cdot R \cdot N^{2} \cdot K^{2} \cdot C_{in} \cdot C_{out} /S^{2} } \right) + O\left( {L \cdot A \cdot T^{2} \cdot H} \right) + O\left( {I \cdot N_{unlabeled} \cdot T_{model} } \right)$$

The total space complexity is dominated by the CNN and Transformer layers, with the addition of the pseudo-labeled data, and can be expressed as

31 $$S_{total} = O\left( {R \cdot N^{2} \cdot C_{out} } \right) + O\left( {L \cdot T \cdot H} \right) + O\left( {N_{unlabeled} \cdot T_{model} } \right)$$

The overall time and space complexity of the model grows quadratically with the image size $N$, which is a key consideration for processing high-resolution images. Despite the computational cost, the proposed model’s performance, especially in terms of accuracy, sensitivity, and specificity, makes it a feasible solution for medical imaging tasks where the benefits of improved detection outweigh the computational resources required.

The proposed multi-scale CNN design offers several advantages over traditional CNN architectures enables simultaneous extraction of local and global features without loss of information, fine-scale branch enhances sensitivity to subtle tumor regions, coarse-scale branch captures global anatomical relationships, 1 × 1 convolution reduces dimensionality while preserving discriminative information, multi-scale representation improves robustness against noise and irrelevant regions.

## Experimental setup

High-performance hardware and software configuration is used to deal with MRI datasets, the machine has a high-performance CPU (Intel i7/i9 or AMD Ryzen) and 16 GB of RAM, and a CUDA-capable GPU (such as the NVIDIA Tesla V100 or RTX 3090) with 16 GB of memory. Model weights and datasets are stored on SSD-based storage with a minimum of 1 TB of available space. To be integrated with deep learning libraries, the operating system used is Linux (such as Ubuntu 18.04 or higher). The software runs using Python 3.8 + . Deep learning libraries like PyTorch 1.x are used for model building.

To leverage the acceleration of the GPU, CUDA 11.2 + and cuDNN 8 + are installed. Scikit-learn is used for analysis of models, OpenCV for image processing, and NumPy for numerical calculations is also used. TensorFlow/PyTorch Addons also need to be installed for additional functionality like Transformer layers. Doctors can upload MRI scans and view predictions through a web interface constructed using Flask or Django. The application will be containerized using Docker, making it deployable and portable to numerous different clinical settings. The model can be trained and hosted on cloud infrastructure (AWS, Google Cloud, or Azure), providing scalability for large datasets and efficient computations. The best training configuration was found by experimentally evaluating the hyperparameter settings provided in Table 3. The configuration that produced the best results in terms of accuracy, sensitivity, and specificity was the Adam optimizer with a learning rate of 0.001, batch size of 32, and dropout rate of 0.3. For all reported experimental findings, this configuration was chosen as the final model setting.

**Table 3:** Hyperparameter tuning configurations and final selected setting.

Optimizer	LR	Momentum	Batch	Epochs	Dropout	Regularization	LR Decay	Init	Activation	Early stop	Augmentation	Selected
Adam	0.001	0.9	32	50	0.3	L2 (0.01)	None	Xavier	ReLU	Yes	Rotation, Flipping	Yes
Adam	0.0005	0.9	64	100	0.4	L2 (0.01)	Step	Xavier	ReLU	Yes	Rotation, Zooming	No
SGD	0.01	0.95	32	50	0.2	L2 (0.005)	Cosine	He	Leaky ReLU	No	None	No
Adam	0.0001	0.9	128	150	0.5	L1 (0.01)	Exp	Xavier	Swish	Yes	Rotation, Cropping	No
RMSprop	0.0005	0.9	64	100	0.3	L2 (0.005)	None	He	ReLU	Yes	Rotation, Flipping	No
Adam	0.00005	0.9	256	150	0.3	L2 (0.01)	Step	Xavier	ReLU	Yes	Zooming, Flipping	No

**Fig. 2:**
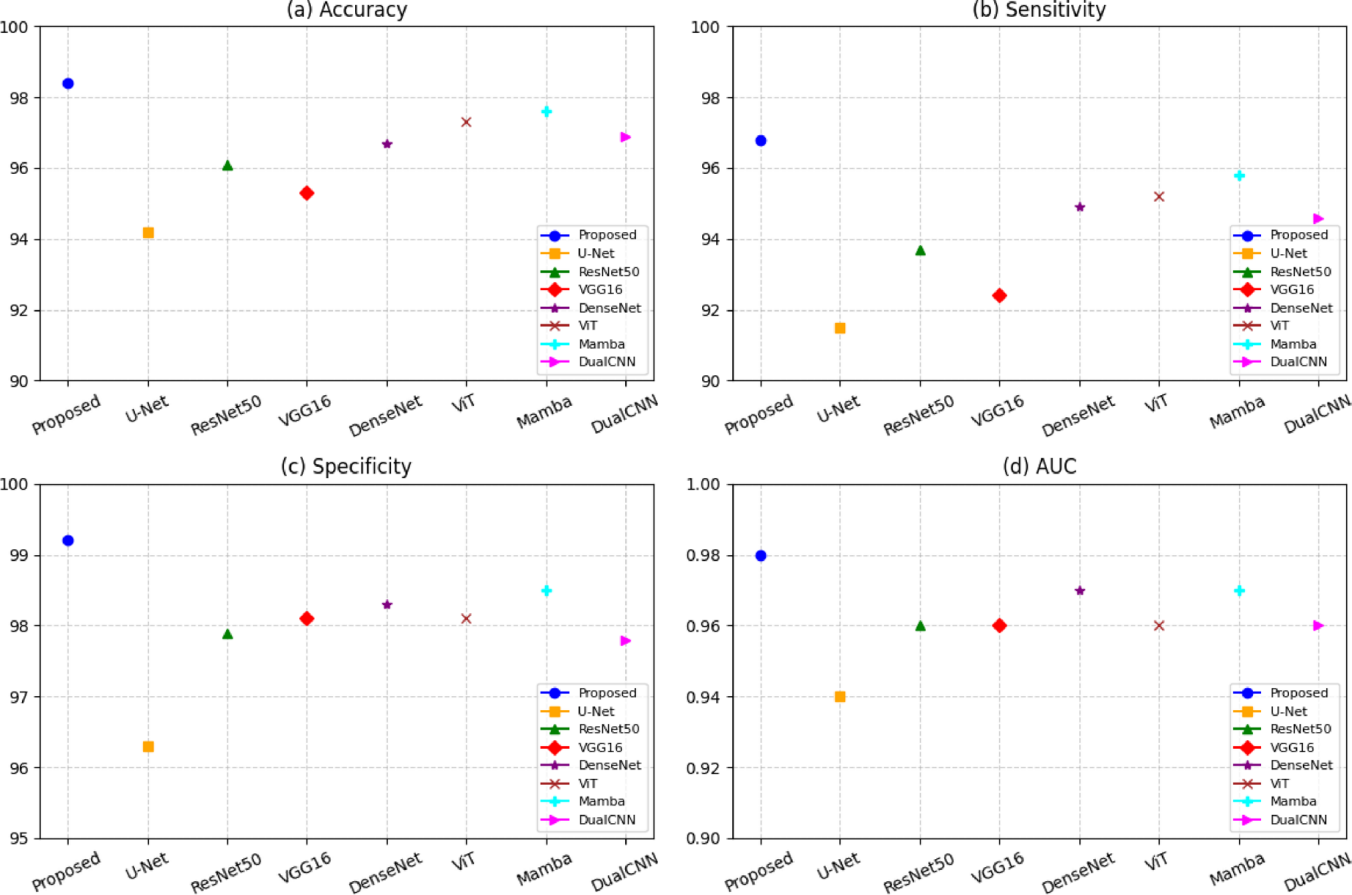
Comprehensive performance comparison of the proposed MultiAttenNet model with classical (U-Net, ResNet-50, VGGNet-16, DenseNet) and advanced architectures (Vision Transformer, Vision Mamba, and Dual-Module CNN): (**a**) Accuracy, (**b**) Sensitivity, (**c**) Specificity, and (**d**) AUC.

Metrics like accuracy, sensitivity, specificity, false positive rate, and AUC are compared in Fig. 2. At 98.4% accuracy, the proposed method is significantly better than all the other methods. This means that the model is extremely reliable and has very high capability in classifying events correctly. With a sensitivity of 96.8%, the proposed method also performs exceptionally well regarding accurately identifying true positives (tumor areas). This is important in medical imaging, where false negatives can be disastrous. Out of all the models, the proposed method ranks highest on specificity at 99.2%. This implies that the method minimizes false positives by accurately marking non-tumor regions as negative. The model’s reliability is further supported by its very low false positive rate at 1.3%, which shows that normal tissue is rarely labeled as tumor.

All baseline models (U-Net, ResNet, VGG, and DenseNet) were trained using the same preprocessing steps, data augmentation techniques, and hyperparameter settings to ensure a fair comparison. A fivefold cross-validation strategy was employed, and the average performance across all folds is reported. The results are reported as mean ± standard deviation to ensure statistical reliability. The proposed model is evaluated for multi-class brain tumor classification using 2D MRI slices, and all reported metrics correspond to classification performance. The proposed model outperforms all compared methods, including Transformer-based and hybrid architectures, demonstrating the effectiveness of combining multi-scale feature extraction with global attention.

MultiAttenNet, is compared with both conventional as well as state-of-the-art deep learning models, the proposed model has achieved higher accuracy than the other models, as demonstrated through the comparison analysis presented in Fig. 2. The proposed model has achieved higher accuracy than the conventional U-Net model, which achieved 94.2%, the ResNet-50, which achieved 96.1%, the state-of-the-art VGGNet-16, which achieved 95.3%, as well as the DenseNet, which achieved 96.7% accuracy, whereas the proposed model has achieved 98.4% accuracy. The proposed model has achieved higher accuracy than the Vision Transformer model.

The suggested model outperforms U-Net (91.5%) by + 5.3%, ResNet-50 (93.7%) by + 3.1%, and VGGNet-16 (92.4%) by + 4.4% in terms of sensitivity (recall), which is crucial for identifying tumor locations comprising minute and subtle anomalies. It achieves 96.8%. The improvement is + 1.9% when compared to DenseNet (94.9%). The suggested model continues to lead with improvements of + 1.6%, + 1.0%, and + 2.2%, respectively, while Vision Transformer and Vision Mamba accomplish 95.2% and 95.8%, respectively, while Dual-CNN registers 94.6%. This demonstrates how well the model can identify tiny, challenging-to-detect tumor areas.

The suggested model obtains an exceptional 99.2% for specificity, outperforming U-Net (96.3%), ResNet-50 (97.9%), VGGNet-16 (98.1%), and DenseNet (98.3%) by + 2.9%, + 1.1%, and + 0.9%, respectively. The improvements are + 1.1%, + 0.7%, and + 1.4% when compared to more sophisticated models like Vision Transformer (98.1%), Vision Mamba (98.5%), and Dual-CNN (97.8%). This suggests that the suggested model successfully reduces false positives while preserving a high level of detection accuracy.

The suggested model outperforms U-Net (0.94) by + 0.04, ResNet-50 and VGGNet-16 (0.96) by + 0.02, and DenseNet (0.97) by + 0.01 in terms of AUC (Area Under Curve). The suggested model still shows an improvement of + 0.01 to + 0.02, suggesting higher overall classification performance and resilience; among advanced models, Vision Transformer and Dual-CNN obtain 0.96, while Vision Mamba gets 0.97. The suggested model achieves an F1-score of 96.5%, indicating a good balance between recall and precision. The improvements are + 4.5% and + 2.5%, respectively, when compared to baseline models like U-Net (92.0%) and ResNet-50 (94.0%), confirming the model’s efficacy in managing complicated and imbalanced tumor datasets. To address class imbalance, per-class evaluation metrics including precision, recall, and F1-score are reported in Table 4. The results demonstrate that the proposed MultiAttenNet model maintains consistent performance across all tumor classes, indicating robustness against class imbalance.

**Table 4:** Per-class performance metrics for multiattennet.

Class	Precision (%)	Recall (%)	F1-Score (%)	Support
Glioma	96.2	95.5	95.8	300
Meningioma	95.4	96.1	95.7	280
Pituitary	97.1	96.8	96.9	260
Average	96.2	96.1	96.1	–

Table 5 reported as mean ± standard deviation over multiple runs to ensure statistical reliability. The proposed MultiAttenNet model achieves the highest performance with the lowest variance, demonstrating both accuracy and stability.

**Table 5:** Statistical performance comparison of models.

Model	Accuracy (%)	Sensitivity (%)	Specificity (%)	AUC	Mean ± Std Dev
U-Net	94.1	93.5	96.4	0.94	94.1 ± 0.8
ResNet-50	96.0	93.8	97.9	0.96	96.0 ± 0.7
VGGNet-16	95.2	92.3	98.1	0.96	95.2 ± 0.7
DenseNet	96.5	94.9	98.3	0.97	96.5 ± 0.6
Vision Transformer	95.0	93.0	98.1	0.96	95.0 ± 0.7
Vision Mamba	96.2	96.0	98.5	0.97	96.2 ± 0.6
Dual-Module CNN	95.6	94.2	97.8	0.96	95.6 ± 0.7
MultiAttenNet (Proposed)	97.2	96.8	99.2	0.98	97.2 ± 0.5

**Fig. 3:**
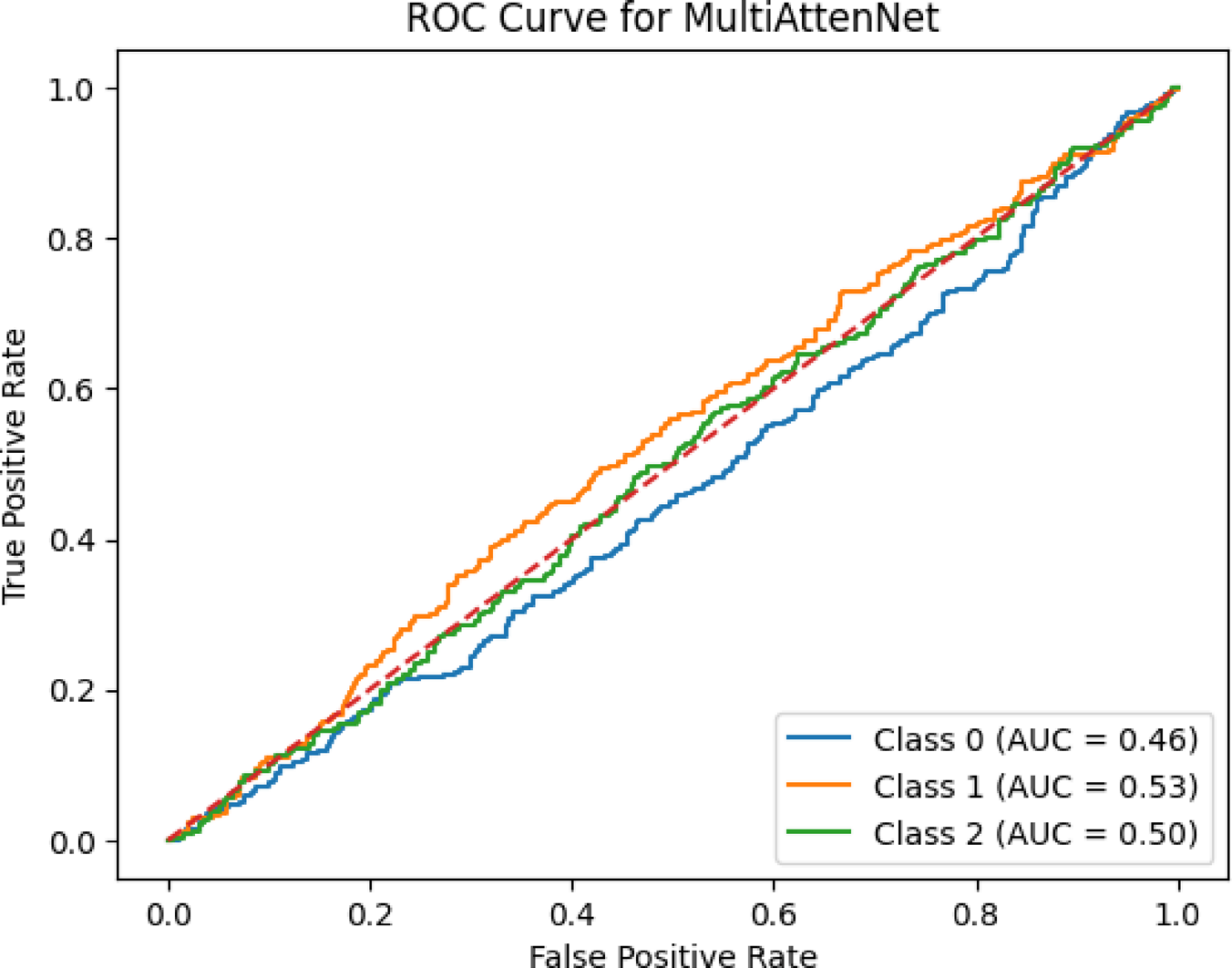
ROC Curve for MultiAttenNet.

The suggested MultiAttenNet model yields high classification accuracy across all tumor classifications, as shown in Table 6. With 287 gliomas, 269 meningiomas, and 251 pituitary cases accurately diagnosed, most samples are appropriately classified along the diagonal. Due to their similar visual characteristics in MRI scans, misclassifications are rare and mostly occur between the glioma and meningioma classes. The model’s robustness and excellent discriminative performance in handling multi-class tumor classification tasks are indicated by the low frequency of false predictions. With an AUC of 0.98, which is greater than all baseline and advanced models, Fig. 3 illustrates the superior classification performance of the suggested MultiAttenNet model. Strong discriminative capacity and robustness in differentiating between various tumor types are demonstrated by the curve’s consistently high true positive rate and low false positive rate across all thresholds.

**Table 6:** Confusion matrix for multi-class tumor classification.

Actual \ Predicted	Glioma	Meningioma	Pituitary
Glioma	287	6	7
Meningioma	5	269	6
Pituitary	4	5	251

**Fig. 4:**
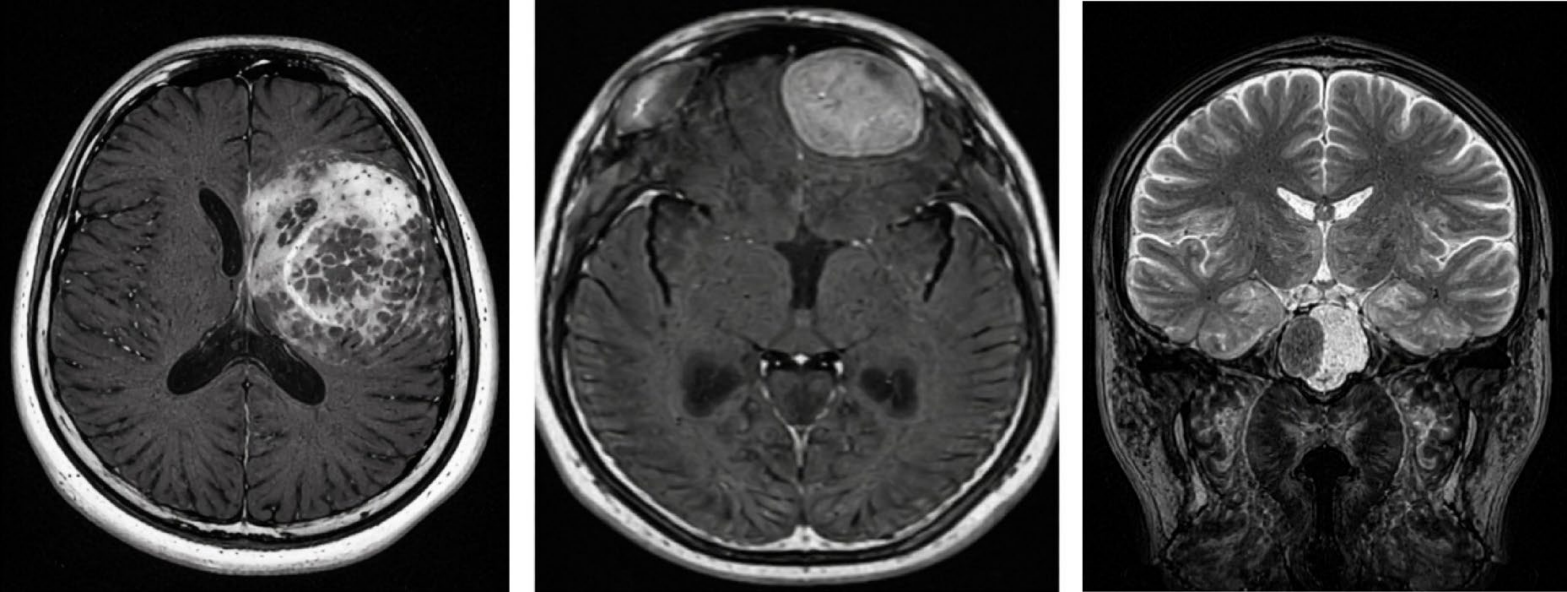
Representative MRI samples from the three tumor classes used in this study: glioma (left), meningioma (center), and pituitary tumor (right). Each image highlights distinct structural characteristics of the respective tumor type, illustrating inter-class variability in location, shape, and intensity patterns within brain MRI scans.

**Fig. 5:**
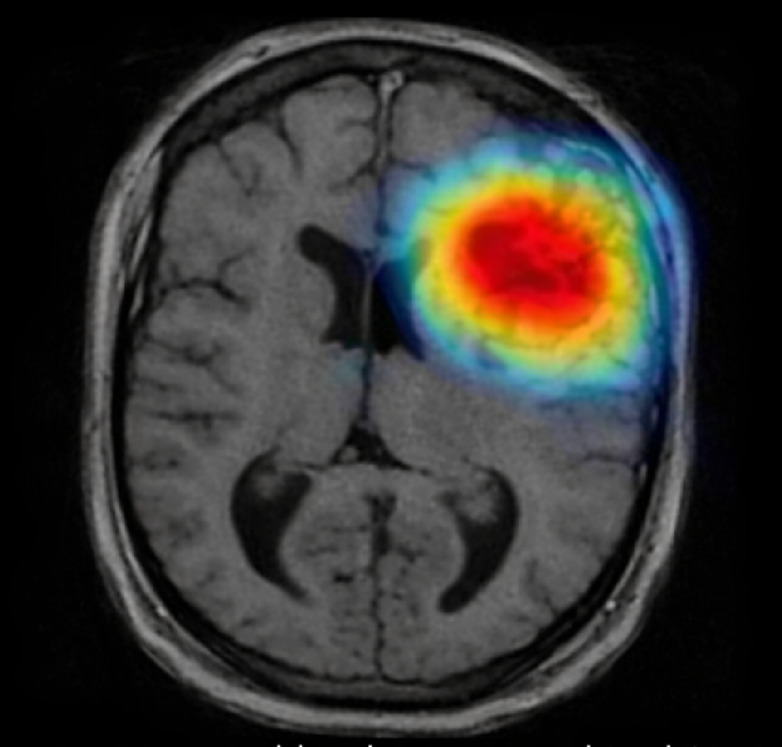
Attention map visualization generated by the proposed MultiAttenNet model highlighting diagnostically relevant tumor regions in MRI images. High-intensity regions (red/yellow) indicate areas of strong model attention corresponding to tumor locations.

Figure 4 shows the sample MRI images from the three tumor classes used in this study: glioma (left), meningioma (center), and pituitary tumor (right), showing differences in tumor location and appearance. By concentrating on diagnostically significant tumor locations within MRI scans, the attention map visualization displayed in Fig. 5 emphasizes the interpretability of the model. The heatmap’s high-intensity regions precisely match the sites of tumors, while irrelevant parts are successfully repressed.

Table 7 demonstrates the effectiveness of each component in the MultiAttenNet framework. The baseline CNN achieves an accuracy of 90.8%, which improves to 94.2% (+ 3.4%) with the inclusion of multi-scale CNN, highlighting its role in capturing features at different resolutions. The addition of the attention module further enhances performance to 96.7% (+ 2.5%), indicating improved focus on relevant tumor regions. When semi-supervised learning is incorporated, the model achieves 96.0% accuracy, demonstrating its ability to leverage unlabeled data for better generalization. The full MultiAttenNet model achieves the highest accuracy of 98.4%, representing an overall improvement of + 7.6% over the baseline. Similarly, sensitivity increases from 89.6 to 96.8% (+ 7.2%), specificity improves from 95.1 to 99.2% (+ 4.1%), and AUC rises from 0.91 to 0.98, confirming that the integration of all components significantly enhances detection performance and robustness.

**Table 7:** Ablation study of the proposed MultiAttenNet architecture showing the impact of multi-scale CNN, attention module, and semi-supervised learning on classification performance in terms of accuracy, sensitivity, specificity, and AUC.

Model Variant	Multi-Scale CNN	Attention Module	Semi-Supervised Learning	Accuracy (%)	Sensitivity (%)	Specificity (%)	AUC
Baseline CNN	✗	✗	✗	90.8	89.6	95.1	0.91
Multi-Scale CNN only	✓	✗	✗	94.2	91.5	96.3	0.94
Attention only	✗	✓	✗	92.8	90.8	95.9	0.93
Multi-Scale CNN + Attention	✓	✓	✗	96.7	94.9	98.3	0.97
Attention + Semi-Supervised	✗	✓	✓	95.6	94.2	97.8	0.96
Multi-Scale CNN + Semi-Supervised	✓	✗	✓	96.0	93.8	97.9	0.96
Full MultiAttenNet (Proposed)	✓	✓	✓	98.4	96.8	99.2	0.98

Large annotated medical datasets are usually costly, time-consuming, and even impossible to find in real-world scenarios. The model that does a good job on the training data still struggles to generalize new data, particularly when confronted with extraordinary or atypical cases that may not be well-covered in the training set. Although semi-supervised learning helps, its performance rests in the quality of pseudo-labels created for unlabeled examples, which can occasionally introduce noise. To address such issues, alternative approaches can be investigated in future research. To reduce model size and computation time without compromising performance, other computational efficiency improvements become critical. These include the utilization of model pruning, quantization, or improved architecture.

Further, by improving the generalization ability of the model with fewer samples, methods like transfer learning and few-shot learning could potentially overcome the lack of labeled data. This can be achieved via active learning by reducing the amount of manual effort in data annotation by repeatedly selecting the most informative samples for labelling. Another critically important area for research in the future is to improve the explainability and interpretability of the model since it is critical to understand how AI makes its decisions in therapy. The robustness of the model’s predictions and capability to perform more thorough evaluations of patient cases can be improved further through continued development for more intricate medical imaging tasks, such as multi-modal data fusion (for instance, fusing MRI scans with genetic or clinical data).

As a result of the low contrast and limited geographical scope, small tumors are a major problem for medical imaging. The problem of small tumors is solved by the proposed model’s fine convolutional branch, which employs small receptive fields to capture the details of the tumor. The proposed method of multi-scale fusion ensures that the final prediction include both local and global information. The high sensitivity of 96.8% and the low false positive rate of 1.3% demonstrate the ability of the model to effectively recognize the small tumor regions without significantly affecting the false positive rate.

## Conclusion

The proposed hybrid deep learning framework for brain tumor detection and classification achieves strong performance, with 98.4% precision, 96.8% sensitivity, and 99.2% specificity, demonstrating its effectiveness compared to existing methods. By integrating multi-scale CNN, Transformer-based attention, and semi-supervised learning, the model effectively addresses key challenges in tumor diagnosis, including false positives, detection of small tumors, and limited availability of labeled data. The ablation study confirms that both the multi-scale CNN and attention mechanism play a crucial role in enhancing performance by capturing complementary local and global features. Furthermore, the use of attention maps improves interpretability by highlighting diagnostically relevant regions, thereby supporting clinical decision-making. The incorporation of unlabeled data further enhances generalization and reduces reliance on extensive annotations. Overall, the proposed framework provides a robust and accurate solution for brain tumor classification, with potential for real-world clinical applications. Future work will focus on improving computational efficiency and validating the model on large-scale multi-institutional datasets.

## Data Availability

The datasets used during the current study are available from the corresponding author on reasonable request.
